# MicroRNAs in Cardiac Hypertrophy

**DOI:** 10.3390/ijms20194714

**Published:** 2019-09-23

**Authors:** Nadine Wehbe, Suzanne A. Nasser, Gianfranco Pintus, Adnan Badran, Ali H. Eid, Elias Baydoun

**Affiliations:** 1 Department of Biology, American University of Beirut, P.O. Box 11-0236, Beirut, Lebanon; nww04@mail.aub.edu; 2 Department of Pharmacology and Therapeutics, Beirut Arab University, P.O. Box 11-5020, Beirut, Lebanon; san413@bau.edu.lb; 3 Department of Medical Laboratory Sciences, University of Sharjah, P.O. Box 27272, Sharjah, UAE; gpintus@sharjah.ac.ae; 4 Department of Nutrition, University of Petra, P.O. Box 961343, Amman 11196, Jordan; abadran@uop.edu.jo; 5 Department of Pharmacology and Toxicology, American University of Beirut, P.O. Box 11-0236, Beirut, Lebanon; 6 Department of Biomedical Sciences, Qatar University, P.O. Box 2713, Doha, Qatar

**Keywords:** cardiac remodeling, cardiac hypertrophy, MicroRNAs, therapeutic targets, cardiomyocyte

## Abstract

Like other organs, the heart undergoes normal adaptive remodeling, such as cardiac hypertrophy, with age. This remodeling, however, is intensified under stress and pathological conditions. Cardiac remodeling could be beneficial for a short period of time, to maintain a normal cardiac output in times of need; however, chronic cardiac hypertrophy may lead to heart failure and death. MicroRNAs (miRNAs) are known to have a role in the regulation of cardiac hypertrophy. This paper reviews recent advances in the field of miRNAs and cardiac hypertrophy, highlighting the latest findings for targeted genes and involved signaling pathways. By targeting pro-hypertrophic genes and signaling pathways, some of these miRNAs alleviate cardiac hypertrophy, while others enhance it. Therefore, miRNAs represent very promising potential pharmacotherapeutic targets for the management and treatment of cardiac hypertrophy.

## 1. Introduction

The heart undergoes adaptive remodeling with age, which is exacerbated by stress and pathological stimuli, subsequently leading to cardiovascular diseases [1,2]. Cardiac hypertrophy is a form of remodeling characterized by the enlargement of cardiomyocytes without an increase in their number. As the heart increases in size, the ventricles are enlarged, and the workload pressure on the ventricular walls decreases [1,3,4]. This helps in maintaining cardiac output efficiency under stress and pathological conditions.

Hypertrophy is classified as a physiological or pathological process, depending on the stimuli and the underlying molecular mechanisms [4,5]. Physiological hypertrophy normally occurs during pregnancy, infant growth, and athletic activities [6]. On the other hand, pathological hypertrophy, which if untreated can lead to heart failure and death, is triggered by conditions including hypertension, myocardial infarction, obesity, and diabetes [7]. Hallmarks of pathological cardiac hypertrophy include apoptosis, fibrosis, and the upregulation of fetal cardiac gene expression [7,8]. Based on the morphology of cardiomyocytes, cardiac hypertrophy is characterized as concentric or eccentric. Concentric cardiac hypertrophy is defined as a reduction in ventricular dimension and an increase in cardiomyocyte thickness-to-length ratio, whereas in eccentric hypertrophy, cardiomyocytes are lengthened and the ventricular chamber is widened [9,10].

Cardiac hypertrophy is a target of several therapeutic approaches, including pharmacological, dietary supplements, and stem cell and cardiac regeneration, as well as RNA-based therapies [11,12]. A particular RNA-based therapy using microRNAs has gained attention in recent years, due to its regulation of both cardiac physiology and pathology [13,14]. Although some microRNAs exhibit a therapeutic role against cardiac hypertrophy, others have been shown to promote hypertrophy [15].

## 2. MicroRNAs (miRNAs)

MicroRNAs (miRNAs) are small, non-coding RNAs approximately 20 to 22 nucleotides in length. They are vital regulators of several intracellular signaling pathways, which might explain why they are evolutionary conserved. They play a master role in regulating gene expression. Through an imperfect base pairing with their target mRNA, a single miRNA can consequently regulate the expression of more than 100 different transcripts, thus altering up to 60% of protein-encoding genes at the translational level [16,17,18]. MiRNAs originate from long primary transcripts, which are cleaved in the nucleus by Drosha, a class 2 ribonuclease III, into miRNA precursors (pre-miRNAs) [19,20]. Pre-miRNAs are then transported to the cytoplasm, to be cleaved by the endonuclease Dicer forming double-stranded miRNAs [21]. One of the strands, known as the guide strand, binds to an RNA-induced silencing complex (RISC), which uses it to bind to the target gene by base pairing [22].

**Figure 1:**
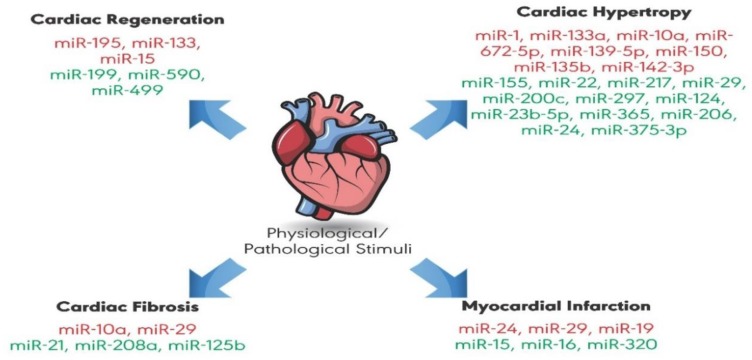
MicroRNAs (miRNAs) play a role in physiological and pathological cardiac remodeling. Some miRNAs have a protective role against cardiovascular diseases, whereas others promote extensive cardiac remodeling, leading to disease. The red color indicates miRNAs that negatively regulate cardiac remodeling. The green color indicates miRNAs having a positive regulatory role.

Through studying miRNA–3′untranslated region (UTR) interactions or gain/loss-of-function, miRNAs were found to regulate the expression of genes related to cardiovascular disease. In this regard, different miRNAs have been implicated in cardiac arrhythmia [23], myocardial infarction [24,25,26], valvular heart disease [27], and genetically inherited cardiomyopathy [28,29]. In addition, it is well established that miRNAs are involved in different routes of cardiac remodeling (Figure 1) [30,31,32,33,34,35]. MiRNAs act as positive or negative regulators of cardiac hypertrophy by targeting pro-hypertrophic signaling pathways, such as calcium signaling and cell cycle-related pathways. In our review, we aim to focus on majorly studied anti-hypertrophic and pro-hypertrophic miRNAs, as well as recently investigated miRNAs, by reviewing “microRNAs or miRNAs and cardiac hypertrophy” related articles on PubMed. We describe the role of miRNAs and their dysregulation in mediating cardiac hypertrophy. We also provide a detailed and critical discussion of their targeted pathways and the underlying mechanisms involved in cardiac hypertrophy, with the goal of uncovering the incremental insights into their potential as therapeutic agents.

## 3. MicroRNAs that Attenuate Cardiac Hypertrophy

### 3.1. MiR-1

MiR-1, a muscle-specific miRNA, is abundantly expressed in the heart, and plays a protective role against cardiac hypertrophy by targeting a number of pro-hypertrophic signaling pathways [36].

**Figure 2:**
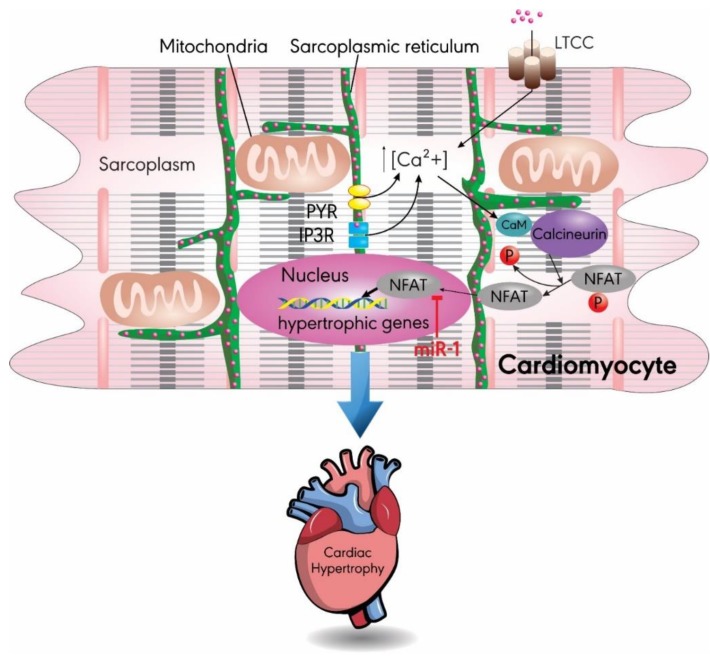
MiR-1 targets the calcineurin–NFAT signaling pathway; miR-1 plays a protective role in cardiac hypertrophy by decreasing the expression of NFAT. An increase in intracellular calcium ion levels activates the CaM–calcineurin complex, which dephosphorylates NFAT, leading to its translocation into the nucleus. In the nucleus, expression of NFAT increases the transcription of hypertrophic genes, resulting in cardiac hypertrophy. CaM: calmodulin; IP3R: inositol 1,4,5-triphosphate receptor; LTCC: L-type calcium channel; RYR: ryanodine receptor; SR: sarcoplasmic reticulum.

Calcium signaling is a known pro-hypertrophic pathway, where an increase in intracellular Ca^2+^ levels acts as a signal for hypertrophy by increasing cardiac output [37]. The calcium-dependent serine/threonine protein phosphatase, calcineurin, induces hypertrophy by acting through the transcription factor nuclear factor of activated T cells (NFATC) 3 [38,39,40]. In human heart tissue samples and mice cardiomyocytes, overexpression of miR-1 ameliorated cardiac hypertrophy by decreasing the expression NFATC3. This was confirmed by a reduction in the cell surface area of cardiomyocytes and a decrease in the levels of beta-myosin heavy chains (β-MHCs), a marker of cardiac hypertrophy (Figure 2) [41,42].

Another study investigated the role of miR-1 in cardiac hypertrophy by targeting mitochondrial calcium uniporter (MCU), the pore-forming subunit of the mitochondrial Ca^2+^ uniporter complex (MCUC). MCUC serves a major role in cardiomyocytes stress adaptation by controlling Ca^2+^ uptake in the mitochondria. Analysis of human heart biopsies from patients with cardiac hypertrophy revealed that a decrease in miR-1 levels was associated with an increase in MCU protein content [43].

MiR-1 can also protect the heart against hypertrophy by regulating the cyclin D kinase 6-retinoblastoma (CDK6-Rb) pathway and levels of thyroid hormone (TH) [44,45]. Hypertrophic rat cardiomyocytes transfected with miR-1 mimics or CDK6 siRNA inhibit activation of the CDK6-Rb pathway, which is an important pathway in the regulation of cell cycle progression contributing to cardiac hypertrophy [44,46].

In a recent study, Diniz et al. [45] report that overexpression of miR-1 prevented TH-induced cardiac hypertrophy, as indicated by decreased levels of atrial natriuretic peptide (ANP) and alpha-myosin heavy chain (α-MHC), two markers of cardiac hypertrophy. In addition, TH was found to induce hypertrophy by targeting histone deacetylase-4 (HDAC4). Indeed, overexpression of miR-1 reduced the expression of HDAC4, and inhibition of HDAC4 gene helped attenuate TH-induced cardiac hypertrophy [45].

Downstream targets of miR-1 include the cytoskeleton regulatory protein twinfilin-1 (TWF1) [47]. TWF1 regulates actin dynamics by binding actin monomers, which are known to control various cell biological processes, such as motility, endocytosis, cell division, and signal transduction [48]. Interestingly, the protein level of TWF1 is inversely related to the expression pattern of miR-1 [47]. Given that miR-1 negatively regulates cardiac hypertrophy [36], rat cardiomyocytes overexpressing miR-1 displayed reduced cell size and suppressed TWF1 protein expression [47]. Conversely, hypertrophic rat hearts and phenylephrine (PE)-induced hypertrophic cardiomyocytes showed miR-1 downregulation, which was associated with upregulated TWF1 and actin protein levels [47].

Insulin-like growth factor-1 (IGF-1) was also reported to be among miR-1 cardiac targets mediating cardiac hypertrophy [49]. IGF-1 is known to regulate cardiomyocyte size and contractile function [50]. In the transverse aortic constriction (TAC) model [49] and Akt-transgenic animal model [51], repression of miR-1 was accompanied by an increased IGF-1 protein level and its receptor, IGF-1R. This observation was reinforced with the clinical demonstration of depressed miR-1 levels in biopsies of patients with acromegaly, a condition characterized by the overproduction of growth hormone and IGF-1, as well as increased cardiac myocyte size [49].

### 3.2. MiR-133a

Similar to miR-1, miR-133a is a muscle-specific miRNA, which exerts an anti-hypertrophic effect through offsetting multi-targets involved in the calcium signaling, cell growth, and cell development pathways [52,53,54,55,56,57]. In this regard, miR-133a transfection reduced cardiac hypertrophy in vivo and in vitro by downregulating the mRNA and protein expression of calcineurin, a key player in intracellular Ca^2+^ regulation [52]. Moreover, miR-133a transfection blocked PE-induced cardiomyocyte hypertrophy by downregulating the mRNA and protein levels of calcineurin downstream effector NFATC4 [53]. The anti-hypertrophic effect of miR-133a was further revealed in the TH-induced hypertrophy rat model. Indeed, decreased levels of miR-133 were reported in this animal model, and were thought to be partially mediated by angiotensin II receptor subtype 1 (AT1R) [57]. Furthermore, this AT1R appears to also play a role in increasing the expression of two miR-133 targets, namely calcineurin and sarcoplasmic/endoplasmic reticulum Ca^2+^ ATPase 2a (SERCA2a) [57]. Taken together, these reports support a role for miR-133 in TH-induced cardiomyocyte hypertrophy.

**Figure 3:**
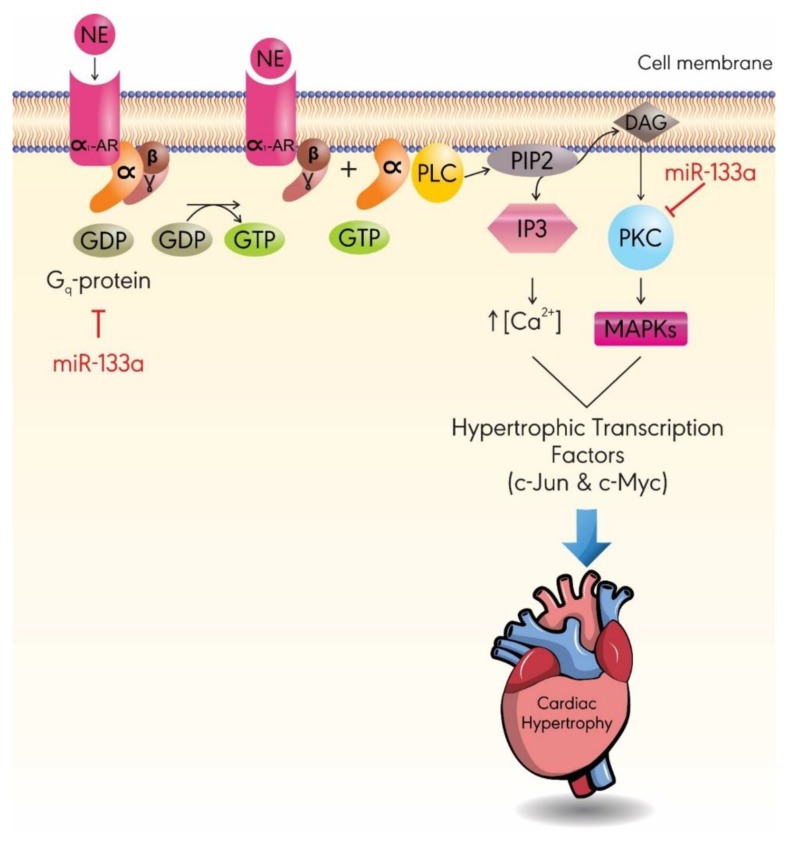
The anti-hypertrophic role of miR-133a is mediated by inhibition of G_q_-protein and PKC pathways. Norepinephrine (NE) binds to α1-adrenergic receptor (α1-AR). Upon its activation, α1-AR couples to G protein, resulting in the activation of PLC. PLC catalyzes the breakdown of Phosphatidylinositol 4,5-bisphosphate (PIP2) into IP3- and DAG-activating calcium signaling pathways, as well as a PKC–MAPK pathway, respectively. Both pathways increase the expression of hypertrophic transcription factors. DAG: diacylglycerol; GDP: guanosine diphosphate; GTP: guanosine-5’-triphosphate; IP3: inositol 1,4,5-triphosphate; MAPK: mitogen-activated protein kinase; PKC: protein kinase C; PLC: phospholipase C.

Recent evidence hints towards a potential interplay between miR-133a and α1-adrenergic receptor (AR)-mediated signaling, thereby affecting calcium-signaling. Indeed, miR-133a significantly inhibits norepinephrine (NE)-induced cardiac hypertrophy in vitro by downregulating levels of protein kinase C (PKC) and G_q_ protein [54]. As depicted in Figure 3, NE binds to α1-AR, which activates the PKC and phospholipase C (PLC) signaling pathway. PKC leads to the activation of transcription factors involved in cardiac hypertrophy through a mitogen-activated protein kinase (MAPK) cascade, whereas PLC signaling products—inositol 1,4,5-triphosphate (IP3) and diacylglycerol (DAG)—activates the calcium signaling pathway and PKC, respectively [58,59,60]. Therefore, by targeting PKC and G_q_, miR-133a can inhibit the increase in intracellular Ca^2+^ levels and the subsequent activation of hypertrophic transcription factors, such as c-Jun and c-Myc.

MiR-133a can also attenuate cardiac hypertrophy by repressing the expression of serum response factor (SRF) and cyclin D2, both of which exacerbate aberrant cardiomyocyte proliferation and cardiac dysfunction [55]. Furthermore, others targets of miR-133a include the cardiogenic transcription factor myocyte enhancer factor 2 (MEF2), as well as serum- and glucocorticoid-responsive kinase-1 (SGK1) and IGF-1R [56]. Contextually, glucose-induced cardiomyocyte hypertrophy produced upregulation of MEF2, SGK1, and IGF-1R, as well as reduced expression of miR-133a. Upregulation of the hypertrophy-associated signaling molecules was attenuated by miR-133a mimics, indicating that MEF2, SGK1, and IGF-1R act downstream of miR-133a [56].

### 3.3. Others

**Figure 4:**
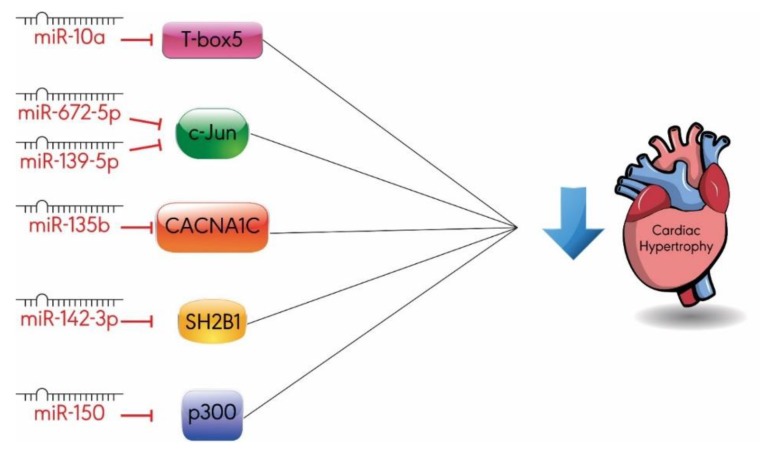
Anti-hypertrophic miRNAs ameliorate cardiac hypertrophy by targeting several biological pathways: miR-10a decreases the expression of T-box5; miR-672-5p and miR-139-5p inhibit c-Jun expression; miR-135b downregulates CACNA1C (L-type calcium channels); miR-142-3p inhibits SH2B1; miR-150 represses the expression of p300. SH2B1: Src homology 2 B adaptor protein 1.

Other miRNAs may also protect the heart against hypertrophy; however, limited research has been conducted to investigate their anti-hypertrophic role (Figure 4). Some of these miRNAs mediate their effect by regulating factors involved in cardiac development or factors involved in calcium signaling and energy metabolism [61,62,63]. Further research is warranted before the roles of the miRNAs can be firmly established. Here, we provide a brief summary of the limited findings pertaining to the role of these miRNAs in cardiac hypertrophy.

## 4. MicroRNAs that Promote Cardiac Hypertrophy

### 4.1. MiR-155

**Figure 5:**
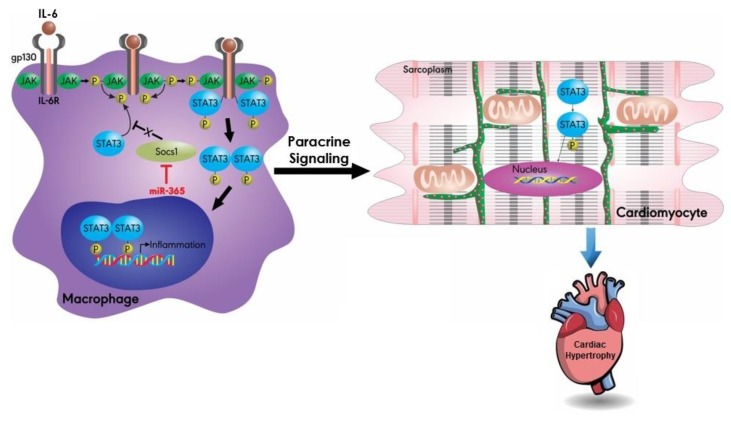
MiR-155 targets the JAK/STAT pathway. The macrophage-expressed miR-155 silences SOCS1 and alleviates its negative regulation of STAT3 phosphorylation. Phosphorylated STAT3 results in inflammation, leading to pro-hypertrophic paracrine signaling to nearby cardiomyocytes. Activation of STAT3 in cardiomyocytes promotes hypertrophy. JAK: Janus kinase; STAT: signal transducer and activator of transcription; IL-6: interleukin 6; IL-6R: interleukin 6 receptor; SOCS1: suppressor of cytokine signaling 1; gp130: glycoprotein 130.

MiR-155 promotes cardiac hypertrophy by targeting pro-hypertrophic pathways, such as inflammation and calcium signaling [64,65,66]. In one study, miR-155-expressing macrophages appeared to promote cardiac hypertrophy through the Janus kinase (JAK)/signal transducer and the activator of the transcription 3 (STAT3) pathway [64]. It has been proposed that the macrophage-expressed miR-155 induces the inhibition of the macrophage suppressor of cytokine signaling 1 (SOCS1), leading to the phosphorylation of STAT3 [67]. This phosphorylated STAT3 in macrophages then drives pro-hypertrophic paracrine signaling to cardiomyocytes, whose STAT3 activity gets potentiated, thereby leading to cardiac hypertrophy (Figure 5) [64].

MiR-10a inhibits cardiac hypertrophy by downregulating the expression of T-box5, a transcription factor involved in cardiac development [61]. Both miR-672-5p and miR-139-5p ameliorate cardiac hypertrophy in cardiomyocytes by inhibiting the expression of another transcription factor c-Jun, a subunit of activator protein-1 (AP-1) [68,69]. Similarly, miR-150 mimics were shown to abrogate glucose-induced cardiomyocyte hypertrophy by repressing the expression of p300, a cofactor for various hypertrophy-responsive transcription factors [70].

As mentioned earlier, Ca^2+^ influx promotes cardiac hypertrophy by increasing cardiac contractions and overload. In particular, L-type Ca^2+^ channels, encoded by CACNA1C, play a role in the hypertrophic signaling pathway. The transfection of hypertrophic cardiomyocytes with miR-135b mimics downregulated CACNA1C mRNA and protein levels and inhibits cardiac hypertrophy, compared to the control group [62].

Intracellular Ca^2+^ levels are also regulated by normal mitochondrial function, which is the key regulator of energy metabolism. MiR-142-3p-attenuated cardiac hypertrophy is overexpressed by inhibiting the expression of Src homology 2 B adaptor protein 1 (SH2B1), a regulator of energy metabolism with a pro-hypertrophic role [63]. Interestingly, miR-142-3p overexpression in cardiomyocytes protects mitochondrial function by increasing mitochondrial density and membrane potential [63]. Although SH2B1 was detected as a direct target for miR-142-3p in cardiac hypertrophy, its role in miR-142-3p-induced mitochondrial protection is unclear. Because mitochondrial dysfunction plays a role in promoting cardiac hypertrophy and other cardiac disorders, it is important to address the protective role of miR-142-3p and other miRNAs in mitochondrial function.

On the other hand, miR-155 contributes to the calcineurin-dependent cardiac hypertrophy pathway, evident by the reduced heart sizes and β-MHC expression levels in calcineurin/miR155 knockout mice [65]. Indeed, miR-155-dependent cardiac hypertrophy was found to be mediated by inhibiting the expression of jumonji AT-rich interactive domain 2 (Jarid2), a key transcriptional regulator of cardiac development and function with histone demethylase activity [65]. Not surprisingly, mice that are homozygous knockout for the jumonji gene exhibited defective expression of atrial natriuretic factor, a hallmark of cardiac hypertrophy [71]. A previous study demonstrated the role of jumonji in cardiac hypertrophy, by linking it to cell proliferation [72]. Indeed, jumonji represses cell proliferation by downregulating the expression of the cyclin *D1* gene of the cell cycle machinery [72]. Taken together, one can speculate that Ca^2+^-dependent pathways and the cell cycle have an interrelated role in miR-155-related cardiac hypertrophy.

Given that angiotensin II (Ang II) promotes cardiac hypertrophy by binding to cardiac AT1R and targeting the calcineurin pathway [73], the contribution of miR-155 in the calcineurin-dependent cardiac hypertrophy pathway was further explored in the Ang II-induced hypertrophy setting. Transfecting cardiomyocytes with miR-155 inhibitors followed by Ang II treatment resulted in an increase in the levels of AT1R, intracellular calcium, and calcineurin beta; however, the levels of cardiac hypertrophic markers, β-MHC and ANP, were not altered [66]. Since miR-155 inhibitors did not decrease hypertrophy, it has been suggested that inhibition of miR-155 and activation of a calcium signaling pathway may lead to the apoptosis of some myocardial cells, leading to a reduction of the levels of myocardial hypertrophy markers [66]. Further research is required to confirm miR-155/calcineurin-induced cardiac hypertrophy.

### 4.2. MiR-22

MiR-22 is abundantly expressed in cardiac and skeletal muscles, and it is upregulated during myocyte differentiation and cardiac hypertrophy [74]. Similar to miR-155, miR-22 can mediate its pro-hypertrophic activity by targeting the calcineurin pathway. In addition, cardiomyocytes transfected with miR-22 inhibitors or isolated from miR-22 knockout mice expressed an increase in the levels of HDAC4 and sirtuin 1 (SIRT1) [74]. Both HDAC4 and SIRT1 have protective roles in relation to cardiac hypertrophy [75,76]. Therefore, one could conclude that miR-22 induces hypertrophy by targeting these proteins, but this is yet to be confirmed.

One of the key pathways implicated in cardiac growth, as well as the promotion of physiological and pathological hypertrophy, is the phosphatase and tensin homolog (PTEN). PTEN is a negative regulator of the phosphatidyl-3 kinase (PI3K)/Akt/mammalian target of the rapamycin (mTOR) pathway, an important player in cardiac function [77]. Studies have shown that miR-22-induced hypertrophy could involve modulation of PTEN levels. Indeed, overexpression of miR-22 in hypertrophic cardiomyocytes decreases PTEN protein levels, and increases cell surface area and the expression of cardiac hypertrophy markers [78]. This negative correlation between miR-22 and PTEN in cardiac hypertrophy was confirmed in another study, which also showed that treatment with atorvastatin, a cholesterol-lowering drug with an anti-hypertrophic role, reversed the effects of miR-22 overexpression by upregulating levels of PTEN [79]. However, these effects of atorvastatin in human patients with cardiac hypertrophy remain lacking.

### 4.3. MiR-217

MiR-217 seems to share PTEN with miR-22 with regard to mediating its hypertrophic effect. In this respect, it was shown that miR-217 overexpression-induced cardiac hypertrophy was counteracted by restoring PTEN expression, as evident by the decreased hypertrophic markers, ANP and β-MHC [80].

Increased expression of miR-217 has also been shown to be associated with pathological cardiac hypertrophy, with the implication of euchromatic histone–lysine N-methyltransferases EHMT1 and EHMT2 [81]. Pathological hypertrophic cues, such as abdominal aortic banding in rats, provoked augmentation of miR-217 expression and a subsequent reduction in mRNA expression of EHMT1/2, essential for prenatal heart development and catalysis of histone 3 lysine 9 dimethylation (H3K9me2) [82]. The concomitant reductions in EHMT1/2 and H3K9m2 triggers re-expression of fetal-associated transcripts, leading ultimately to pathological hypertrophy. Suppression of miR-217 activity prevents the loss of EHMT1/2 and reverses the induction of hypertrophy [81]. This suggests that miR-217, by virtue of its ability to modulate methylation, represents an attractive pharmaco-target in the management/treatment of cardiac hypertrophy.

### 4.4. MiR-29

MiR-29 is another pro-hypertrophic miRNA that is positively correlated with cardiac fibrosis [35]. Indeed, inhibition of or genetic deficiency in miR-29 prevents TAC-induced cardiac hypertrophy, whereas overexpressing miR-29 promotes PE-induced cardiomyocyte hypertrophy [83]. MiR-29-induced cardiac hypertrophy appears to be mediated via indirectly activating the Wnt signaling pathway through the repression of four inhibitory factors, namely GSK3B, ICAT/CTNNBIP1, HBP1, and GLIS2 [83].

Like miR-22 and miR-217, miR-29 has been profiled as a PTEN-targeting miRNA [84]. MiR-29 upregulation, provoked by TAC- and AngII-induced models of cardiac hypertrophy, mitigates PTEN expression. Because PTEN is a negative regulator of the autophagic PI3K/AKT/mTOR cascade, it has been concluded that miR-29-associated PTEN suppression activates the PI3K/AKT/mTOR system, thereby abrogating autophagy and promoting cardiac hypertrophy [84].

In contrast, miR-29 has been reported to exert a cardioprotective effect in isoproterenol-induced cardiac hypertrophy, through the inhibition of a nuclear receptor: peroxisome proliferator-activated receptor δ (PPARδ) [85]. Another study has hinted to a potential role of miR-29a-3p in attenuating endothelin-1-induced cardiomyocyte hypertrophy via inhibiting NFATc4 expression. However, this study was an in vitro study of H9c2 cells [86]. Therefore, further studies are needed to better delineate the cardiac remodeling role of miR-29.

### 4.5. MiR-200c

**Figure 6:**
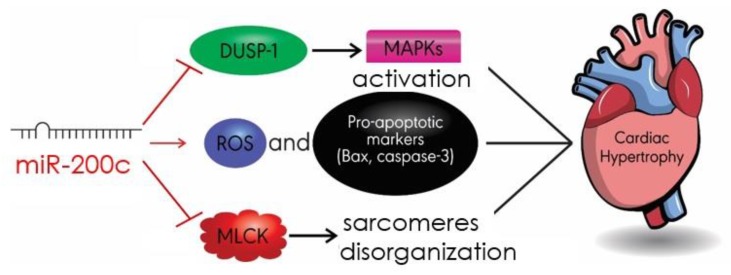
MiR-200c exhibits its pro-hypertrophic role by targeting a number of pathways. Mir-200c reduces the expression of DUSP-1, leading to the activation of MAPKs. MiR-200c targets MLCK and sarcomere organization. MiR-200c can also promote ROS production and enhance the expression of cardiomyocyte pro-apoptotic markers Bax and caspase-3. DUSP-1: dual-specific phosphatase-1; ROS: reactive oxygen species.

Another pro-hypertrophic miRNA is miR-200c, which is implicated in MAPK signaling and reactive oxygen species (ROS)/apoptosis pathways [87,88]. Dual-specific phosphatase-1 (DUSP-1) prevents cardiac hypertrophy by inactivating MAPKs, such as extracellular signal-regulated kinase (ERK1/2), c-Jun N-terminal kinase (JNK), and p38 [89]. Contextually, DUSP-1 was shown to be a target of miR-200c. For instance, miR-200c can induce diabetes-associated cardiac hypertrophy in high-glucose-treated cardiomyocytes, by downregulating the myocardial expression of DUSP-1 and thus activating MAPK proteins (Figure 6) [87].

MiR-200c has also been found to promote cardiac hypertrophy by directly targeting myosin light chain kinase (MLCK), an enzyme involved in cardiovascular physiology and pathophysiology [90]. When miR-200c levels were increased, MLCK levels were reduced [88]. Moreover, in hypertrophic models, overexpression of miR-200c significantly increased ROS production and apoptosis, as indicated by the pro-apoptotic markers Bax and caspase-3 [88]. This is in marked contrast to an earlier study, which suggests that downregulation of miR-200c protects cardiomyocytes from apoptosis [91]. This apparent discordance could be due to the fact that this latter study was performed in H9c2 cells [91], while the former one was performed in rats undergoing aortic banding [88], making its findings more relevant. This calls for further experimentation, to not only study the role of miR-200c in hypertrophy, but also to delineate the role of MLCK in miR200c-induced cardiac hypertrophy, ROS production, and the apoptosis of cardiomyocytes (Figure 6).

### 4.6. Others

**Figure 7:**
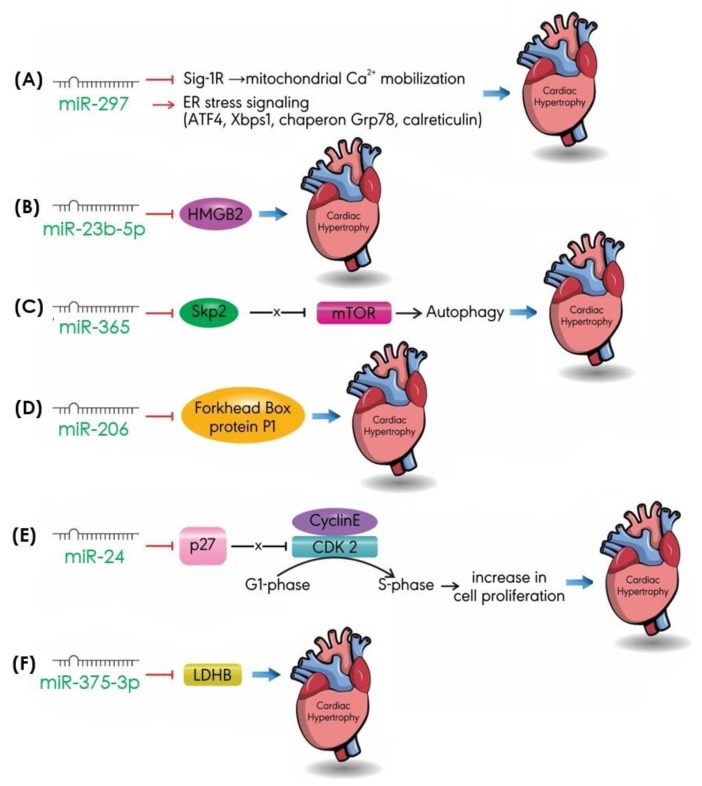
Pro-hypertrophic miRNAs augment cardiac hypertrophy by targeting different pathways: (**A**) miR-297 inhibits the expression of Sig-1R and activates ER stress signaling; (**B**) miR-23b-5p represses HMGB2 expression; (**C**) miR-365 downregulates the expression of Skp2; (**D**) miR-206 negatively regulates the expression of Forkhead box protein P1; (**E**) miR-24 targets p27 and reduces its protein expression; (**F**) miR375-3p abrogates the expression of LDHB. ER: endoplasmic reticulum; HMGB2: high-mobility group box 2; LDHB: lactate dehydrogenase B chain; Sig-1R: sigma-1 receptor; Skp2: S-phase kinase-associated protein 2.

In addition to the miRNAs mentioned above, other, less-studied miRNAs promote cardiac hypertrophy (Figure 7). As noted, an imbalance in the levels of intracellular Ca^2+^ in the heart disrupts the functional homeostasis of the cardiovascular system. Other processes, such as autophagy and cell proliferation, can help maintain functional cardiac homeostasis too [92,93,94].

Intracellular Ca^2+^ levels are regulated by an endoplasmic reticulum (ER) chaperone, sigma-1 receptor (Sig-1R). Inhibition of Sig-1R was shown to damage mitochondrial calcium ion mobilization in cardiomyocytes, thus disrupting intracellular Ca^2+^ levels and promoting cardiac hypertrophy [95]. In vivo and in vitro models of cardiac hypertrophy show upregulation of miR-297, concomitant with the downregulation of Sig-1R [95]. In addition, overexpression of miR-297 was found to increase the protein expression of ER stress markers, such as ATF4, Xbps1, chaperon Grp78, and calreticulin, accelerating the progression of cardiac hypertrophy [96]. MiR-124 is another miRNA that promotes cardiac hypertrophy by increasing the expression of ER stress markers; however, its target gene is yet unknown [92].

A gain-of function study revealed that the hypertrophic effects of miR-23b-5p overexpression observed in the Ang-II- and TAC-induced cardiac hypertrophy models was mediated via targeting high-mobility group box 2 (HMGB2) [97], a nuclear protein that regulates gene transcription, DNA recombination and repair, cell replication, and autophagy [98].

MiR-365 is known as a positive regulator of cardiac hypertrophy; its overexpression leads to an increase in the size of cardiomyocytes [99]. In a recent study, miR-365 was revealed to promote the progression of cardiac hypertrophy by downregulating the expression of S-phase, kinase-associated protein 2 (Skp2), an enzyme involved in the physiological and pathological processes of the heart, such as cardiomyocyte proliferation. Downregulation of Skp2 activates an mTOR signaling pathway, leading to the suppression of autophagy and an increase in cardiac hypertrophy [93]. MiR-206 promoted Yes-associated protein (YAP)-induced cardiac hypertrophy by negatively regulating the expression of Forkhead box protein P1 [100], a transcription factor involved in cell cycle progression, proliferation, and differentiation, as well as in metabolism, survival, and apoptosis [101].

Disruption in the cell cycle and metabolism also promotes cardiac hypertrophy by inducing cell proliferation [102]. In postnatal cardiac cells, proliferation is translated into an increase in size, which leads to hypertrophy, rather than an increase in the cardiomyocyte number [103]. MiR-24 was recently found to induce cardiac hypertrophy by reducing the protein expression of p27, a cell cycle regulator of G_0_/G_1_ arrest [102]. By targeting p27, miR-24 promotes the cells in G_0_/G_1_ phase into S phase, increasing cell proliferation and leading to cardiac hypertrophy [104]. In vitro studies have demonstrated the association between miR-375-3p and cell metabolism. The overexpression of miR-375-3p in cardiomyocytes inhibits protein expression of lactate dehydrogenase B chain (LDHB), a regulator of cell metabolism, and promotes cardiac hypertrophy [94].

## 5. Concluding Remarks

Cardiac hypertrophy is recognized as a risk predictor of sudden cardiac death. Although it is effectively managed by pharmacological interventions, its associated pathophysiological changes are not fully reversed. In addition, experiencing the inevitable treatment-limiting side effects poses higher incidence of cardiovascular events. Emerging evidence provides the basis, and hence the *raison d’etre*, for considering miRNAs as attractive pharmaco-targets in cardiac hypertrophy [105]. The miRNAs tackled in this paper represent the most recently discovered in this field, as well as some of the most studied ones, a list of which is summarized in Table 1. Based on bioinformatics analyses, many in vitro and animal studies have successfully identified downstream targets involved in mediating miRNA regulation of cardiac hypertrophy. However, further studies are needed to examine a detailed analysis of the underlying mechanisms of miRNA-mediated action. Moreover, extrapolation from preclinical findings to clinical practice undoubtedly requires extensive studies on bigger patient cohorts. Additionally, different population traits (age, race, etc.) should be taken into consideration, in order to define the specificity of the miRNA profile before suggesting its candidacy for tailored therapy in cardiac hypertrophy. The blue-sky scenario for miRNAs as potential U.S. Food and Drug Administration (FDA)-approved agents necessitates massive pharmacokinetic testing, which parallels the traditional drug discovery journey. For instance, systemic delivery approaches, (intravenous vs. intracardiac), delivery vehicles, and the dosage and duration of the treatment, should be carefully examined for better target specificity, efficacy, and minimizing off-target effects. Finally, due to its nucleotide-containing nature, the precipitation of the body’s immune response should be strictly investigated.

## Figures and Tables

**Table 1:** Anti- and pro-hypertrophic miRNAs with their targets and the involved signaling pathways.

MiRNAs	Targets	Signaling Pathway	References
Anti-hypertrophic			
miR-1	Calcineurin MCU CDK6-Rb HDAC4 TWF1 IGF-1	Calcium signaling Calcium signaling CDK-Rb pathway Transcription Actin monomer size and localization Transcription	[41] [43] [44] [45] [47] [49]
miR-133a	Calcineurin NFATC4 PLC-PKC SERCA2a SRF/cyclin D2 IGF-1R/SGK1/MEF2	Calcium signaling Calcium signaling Calcium signaling/Transcription Calcium signaling Cell cycle MAPK/ERK	[52] [53] [57] [54] [55] [56]
miR-10a	T-box5	Transcription	[61]
miR-672-5p	c-Jun	Transcription	[68]
miR-139-5p	c-Jun	Transcription	[69]
miR-135b	L-type Ca^2+^ channels	Calcium signaling	[62]
miR-142-3p	SH2B1	Energy balance	[63]
miR-150	p300	Transcription	[70]
Pro-hypertrophic			
miR-155	SOCS1 Jarid2 AT1R	JAK/STAT3 Calcineurin Calcium signaling	[64] [65] [73]
miR-22	HDAC4/SIRT1 PTEN	Calcineurin PI3K/Akt/mTOR	[74] [78,79]
miR-217	PTEN H3K9me2/EHMT1 &2	PI3K/Akt/mTOR Transcription	[80] [81]
miR-29	GSK3B, ICAT/CTNNBIP1, HBP1, GLIS2 PTEN	Wnt signaling PI3K/Akt/mTOR	[83] [84]
miR-200c	DUSP-1 MLCK Bax/cleaved caspase3	MAPK/JANK/p38 Apoptosis	[87] [88] [88]
miR-297	Sig-1R ATF4, Xbps1, Chaperon G78, Calreticulin	Mitochondrial Ca^2+^ mobilization ER stress signaling pathway	[96] [96]
miR-124	ER stress markers	ER stress signaling pathway	[92]
miR- 23b-5p	HMGB2	Transcription/autophagy	[97]
miR-365	Skp2	mTOR	[93]
miR-206	Forkhead box protein P1	Transcription	[100]
miR-24	p27	Cell cycle	[104]
miR-375-3p	LDHB	Cell metabolism	[94]
